# IL-17-mediated Bcl-2 expression regulates survival of fibroblast-like synoviocytes in rheumatoid arthritis through STAT3 activation

**DOI:** 10.1186/ar4179

**Published:** 2013-02-20

**Authors:** Seon-Yeong Lee, Seung-Ki Kwok, Hye-Jin Son, Jun-Geol Ryu, Eun-Kyung Kim, Hye-Jwa Oh, Mi-La Cho, Ji Hyeon Ju, Sung-Hwan Park, Ho-Youn Kim

**Affiliations:** 1 The Rheumatism Research Center, Catholic Research Institute of Medical Science, The Catholic University of Korea, Banpo-dong, Seocho-gu, Seoul, 137-701, South Korea; 2 Center for Rheumatic Disease, Division of Rheumatology, Department of Internal Medicine, Seoul St. Mary's Hospital, The Catholic University of Korea, Banpo-dong, Seocho-gu, Seoul, 137-701, South Korea

## Abstract

**Introduction:**

Fibroblast-like synoviocytes (FLSs) are a major cell population of the pannus that invades adjacent cartilage and bone in rheumatoid arthritis (RA). The study was undertaken to determine the effect of interleukin-17 (IL-17) on the survival and/or proliferation of FLSs from RA patients and to investigate whether signal tranducer and activator of transcription 3 (STAT3) is implicated in this process.

**Methods:**

Bcl-2 and Bax expression in FLSs was determined using the real-time PCR and western blot analysis. The expression of Bcl-2 and phosphoSTAT3 in synovial tissues was investigated by confocal microscope. Apoptosis of FLSs was detected by Annexin V/propidium iodide staining and/or phase contrast microscopy. The proliferation of FLSs was determined by CCK-8 ELISA assay.

**Results:**

The pro-apoptotic Bax is decreased and anti-apoptotic Bcl-2 is increased in FLSs from RA patients compared with those from patients with osteoarthritis (OA). IL-17 upregulated the expression of Bcl-2 in FLSs from RA patients, but not in FLSs from OA patients. STAT3 was found to mediate IL-17-induced Bcl-2 upregulation in FLSs from RA patients. Additionally, IL-17 promoted the survival and proliferation of FLSs from RA patients. Most importantly, treatment with STAT3 inhibitor reversed the protective effect of IL-17 on FLSs apoptosis induced by sodium nitroprusside (SNP).

**Conclusions:**

Our data demonstrate that STAT3 is critical in IL-17-induced survival of FLS from RA patients. Therefore, therapeutic strategies that target the IL-17/STAT3 pathway might be strong candidates for RA treatment modalities.

## Introduction

Rheumatoid arthritis (RA) is a multi-systemic autoimmune disease of unknown etiology that is characterized by hyperplastic synovial membrane capable of destroying adjacent articular cartilage and bone [1,2]. Synoviocytes, the major cell population of invasive pannus, actively participate in the inflammatory processes of RA in the rheumatoid synovium [2]. Synoviocytes produce not only various matrix metalloproteinase, main effector molecules for digesting cartilages and bones, but also pro-inflammatory cytokines such as interleukin (IL)-1 and IL-6 [3], and angiogenic factors such as VEGF (vascular endothelial growth factor) [4,5], all of which play major roles in the pathogenesis of RA [6,7]. In addition, rheumatoid synoviocytes proliferate abnormally and have characteristics mimicking tumors, such as a somatic mutation in H-ras [8], p53 [9], and resistance to FAS (TNF receptor superfamily, member 6)-mediated apoptosis [10].

IL-17 is a highly inflammatory cytokine with robust effects on stromal cells, resulting in the production of inflammatory cytokines and recruitment of leukocytes, and this creates a link between innate and adaptive immunity [11]. IL-17 is produced mainly by pro-inflammatory T-helper 17 (Th17) cells and both IL-17 and Th17 cells are deeply implicated with the pathogenesis of RA [7]. When it comes to the effect of IL-17 on synoviocytes, IL-17 increases the migration, chemokine gene expression and invasiveness of synoviocytes. It also inhibits synoviocyte apoptosis and enhances metalloproteinase production leading to cartilage damage [12].

Signal tranducer and activator of transcription 3 (STAT3) is a transcription factor encoded by the *STAT3 *gene [13]. The STAT3 protein exists in a latent form in the cytoplasm. STAT3 become phosphorylated on tyrosine residues upon receptor activation by cytokines such as IL-6 and then forms homo- or heterodimers that translocate to the cell nucleus, where they act as transcription activators. STAT3 plays an essential role in the differentiation of the Th17 cells [14] and it was reported that STAT3 is activated in inflamed synovium of an animal model of RA [15,16].

In the present study, we hypothesized that STAT3, which is the critical transcription factor for the differentiation of Th17 cells, is implicated in IL-17-dependent survival and proliferation of rheumatoid synoviocytes. We found that an imbalance between pro-apoptotic Bax and anti-apoptotic Bcl-2 exists in FLSs from RA patients. In addition, IL-17 upregulated the expression of Bcl-2 in FLSs from RA patients. Moreover, we observed that STAT3 mediated IL-17-induced Bcl-2 upregulation in FLSs from RA patients. Furthermore, IL-17 promoted the proliferation of synoviocytes and rescues them from apoptotic death via the STAT3 pathway. To our knowledge, our study is the first to demonstrate that STAT3 plays a critical role in IL-17-induced survival and proliferation of synoviocytes from RA patients, thus, highlighting the importance of this pathway in the survival and proliferation of synoviocytes, a critical cell population in the rheumatoid synovium.

## Materials and methods

### Isolation and culture of FLSs

Synoviocytes were isolated by enzymatic digestion of synovial tissue specimens obtained from patients with RA and osteoarthritis (OA) undergoing total joint replacement surgery. The tissue samples were minced into 2- to 3-mm pieces and treated for 4 hours with 4 mg/ml of type I collagenase (Worthington, Freehold, NJ, USA) in DMEM at 37°C in 5% CO_2_. Dissociated cells were then centrifuged at 500 g, resuspended in DMEM supplemented with 10% FCS, 2 mM L-glutamine, 100 units/ml of penicillin, and 100 ng/ml of streptomycin, and plated in 75-cm^2 ^flasks. After overnight culture, nonadherent cells were removed, and the adherent cells were cultivated in DMEM supplemented with 10% FCS. The cultures were kept at 37°C in 5% CO_2_, and the medium was replaced every 3 days. The FLSs, from passages three to seven, were seeded in 24-well plates or 60-mm culture dishes in 10% FCS-supplemented DMEM, and then cultivated for 12 hours at 37°C. Informed consent was obtained from all the participating subjects. The study received approval by the Institutional Review Board of Seoul St. Mary's hospital.

### Real time PCR with SYBR Green

Total mRNA was extracted from the FLSs (1 × 10^6 ^cells) using RNAzol-B, according to the manufacturer's recommendations (Biotecx, Houston, TX, USA). Reverse transcription of 2 μg total mRNA was carried out at 42°C using the Superscript Reverse Transcription system (Takara, Shiga, Japan). PCR amplification of complementary DNA (aliquot) was performed by adding 2.5 mM dNTPs, 2.5 units Taq DNA polymerase (Takara), and 0.25 μM sense and antisense primers. The reaction took place in 25 μl of PCR buffer, consisting of 1.5 mM MgCl_2_, 50 mM KCl, and 10 mM Tris HCl (pH 8.3). The following primers were used for each molecule: for Bcl2, 5'-CAG AAT CCT CTG GAA CTT GAG G-3'(sense) and 5'- GGT CTC CGA ATG TCT GGA AG-3'(antisense); for Bax, 5'-GGT GCC TCA GGA TGC G-3' (sense) and 5' -GGA GTC TGT GTC CAC G-3' (antisense): for IL-17RA, 5'-AGA CAC TCC AGA ACC AAT TCC -3' (sense) and 5'-TGG TGG AGA GCA ACT CTA AGA-3' (antisense): for β-actin, 5'-GGA CTT CGA GCA AGA GAT GG-3' (sense) and 5'-TGT GTT GGG GTA CAG GTC TTT G-3' (antisense). Reactions were processed in a DNA thermal cycler (PerkinElmer Cetus, Wellesley, MA, USA) with 30 cycles of 30 seconds of denaturation at 95°C, and 30 of annealing at 60°C, followed by 30 of elongation at 72°C. Results are expressed as the ratio of product to β-actin product.

### Western blot

The protein of Bcl-2 (Santa Cruz Biotechnology, Santa Cruz, CA, USA), Bax (Santa Cruz Biotechnology), *p*-STAT3 (Cell Signaling Technology, Danvers, MA, USA), STAT3 (Cell Signaling Technology, Danvers, MA, USA) and β-actin (Sigma, St. Louis, MO, USA) were measured by Western blot. RA and OA FLSs were incubated with IL-17 and/or STA21 (Santa Cruz Biotechnology) for 12 hours, a whole cell lysate was prepared from about 2 × 10^5 ^cells by homogenization in lysis buffer, and the lysate was centrifuged at 14,000 rpm for 15 minutes. The protein concentration in the supernatant was determined using the Bradford method (Bio-Rad, Hercules, CA, USA). Protein samples were separated on 10% SDS-PAGE and transferred to a nitrocellulose membrane (Amersham Pharmacia, Uppsala, Sweden). The membrane was incubated with 0.1% skim milk in Tris-buffered saline (TBS) with 0.1% Tween 20 (TTBS). The primary antibodies to Bcl-2, Bax, *p*-STAT, STAT3 and β-actin were diluted in 0.1% skin milk/TTBS and incubated for 10 minutes at room temperature. The membrane was washed three times with TTBS, and horseradish peroxidase (HRP)-conjugated secondary antibody was incubated for 10 minutes at room temperature. After washing with TTBS, the hybridized bands were detected using an enhanced chemiluminescence (ECL) detection kit and Hyperfilm-ECL reagents (Amersham Pharmacia).

### Immunohistochemistry

Immunohistochemical staining for Bcl-2 was performed on FLSs. Briefly, the cells were fixed in 10% formalin solution at room temperature. The FLSs were depleted of endogenous peroxidase activity by adding methanolic H_2_O_2 _and were blocked with normal serum for 30 minutes. After an overnight incubation at 4 C with polyclonal anti-Bcl2 (Santa Cruz Biotechnology), the samples were incubated with biotinylated secondary linking antibodies for 40 minutes and then incubated with streptavidin-peroxidase complex (Vector, Peterborough, UK) for 1 hour followed by an incubation with 3,3';-diaminobenzidine (Dako, Glostrup, Denmark) The samples were counterstained with hematoxylin. Samples were photographed with an Olympus photomicroscope (Tokyo, Japan).

### Confocal microscopy

Synovium was snap-frozen in liquid nitrogen and stored at 80°C. Tissue sections (7 μm) were fixed in 4% paraformaldehyde, blocked with 10% goat serum, and stained with anti-Bcl-2- fluorescein isothiocyanate (FITC) (Santa Cruz Biotechnology), 4',6-diamidino-2-phenylindole (DAPI) (eBioscience, San Diego, CA, USA) and *p*-STAT3 705-PE (BD Biosciences) or *p*-STAT3 727-PE (BD Biosciences). Fluorescence images were acquired using an LSM 510 confocal microscope (Zeiss, Jena, Germany).

### Annexin and propidium iodide (PI) stain

FLSs were washed with PBS and then the cells were stained with Annexin-FITC (BD Biosciences, San Jose, CA, USA) and PI (BD Biosciences) for 15 minutes at room temperature. The cells were subjected to flow cytometric analysis (fluorescence activated cell sorter (FACS) caliber, BD Biosciences).

### Cell proliferation analysis

Cell proliferation was determined by a 2-(2-methoxy-4-nitrophenyl)-3-(4-nitropenyl)-5-(2, 4-disulfophenyl)-2H-tetrazolium (CCK-8) kit (Dojindo Laboratories, Kumamoto, Japan) according to the manufacturer's instructions. Briefly, CCK-8 is reduced by dehydrogenases in cells to yield an orange-colored product (formazan) (Hodgkin *et al*., 1990 [17]). The amount of formazan dye generated by the dehydrogenases was directly proportional to the number of living cells. RA FLSs (1 × 10^4 ^cells per well in 0.1% ITSA (insulin-Transferrin Selenium-A)/DMEM media in a 96-well plate) were cultured in 200 μl medium in the presence or absence of IL-17 (1 or 10 ng/ml) for 12 hours. The CCK-8 solution was added to each well, and the cells were incubated for 2 to 3 hours. Absorbance was measured at 450 nm using a microplate reader.

### Apoptosis assay

Caspases 3/7 activity was assayed using the Apo-ONE™ Homogeneous Caspase 3/7 assay (Promega, Madison, WI, USA) according to the manufacturer's instructions. Briefly, equal volumes of DMEM and Apo-ONE™ caspase reagent (1:100 profluorescent substrate and lysis buffer) were added to cells, and the mixture was incubated for 3 hours. Fluorescence (excitation, 499 nm; emission, 512 nm) was measured using a fluorescence plate reader. Background fluorescence was determined by fluorescence from DMEM alone and subtracted from all experimental values.

### Statistical analysis

The experimental values are presented as mean ± SD. Comparisons of numerical data between the groups were performed by Student's *t*-test or Mann-Whitney *U*-test. Values of *P *< 0.05 were considered statistically significant.

## Results

### Bcl-2 overexpression in FLSs from patients with RA

**Figure 1:**
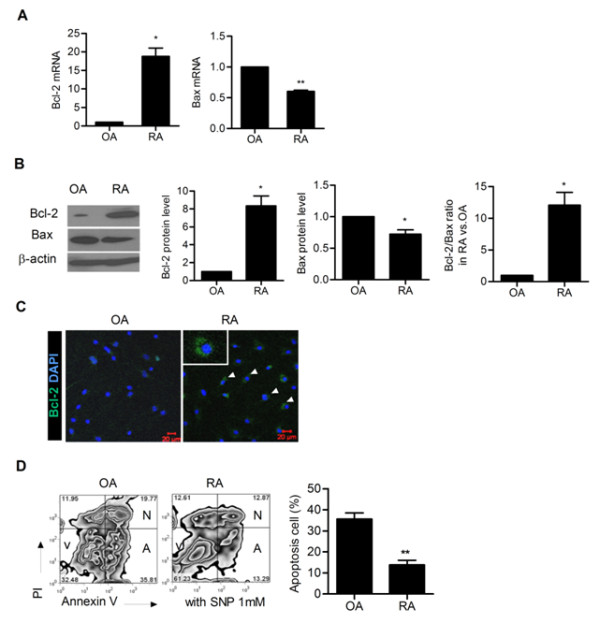
**Bcl-2 and Bax expression in synoviocytes in rheumatoid arthritis (RA)**. (**A**) Fibroblast-like synoviocytes (FLSs) from RA patients (*n *= 3) and osteoarthritis (OA) patients (*n *= 3) were cultured. The expression of Bcl-2 and Bax mRNA in synoviocytes was evaluated by real-time PCR. *P < 0.05, **P < 0.01 compared with OA. (**B**) Expression of Bcl-2 and Bax in synoviocytes was evaluated by western blot. The representative figure is shown in the left panel. The optical density (OD) ratio of Bcl-2 and Bax between FLSs in RA and OA is shown in the right panel. *P < 0.05 compared with OA. (**C**) Expression of Bcl-2 in FLSs in RA and OA was evaluated by confocal microscopy. Bcl-2 expressing FLSs are shown as green. Staining for nuclei by 4',6-diamidino-2-phenylindole (DAPI) is shown as blue. The representative figure is shown. (**D**) Apoptosis was induced by treating the cells with 1 mM sodium nitroprusside (SNP). The degree of apoptosis was assessed by flow cytometry using propidium iodide (PI) and Annexin V. A (apoptotic cells) were defined as PI-Annexin V+ cells. **P < 0.01 compared with OA.

Rheumatoid synoviocytes, the principal components of pannus, proliferate abnormally in a fashion similar to that of a tumor, resist apoptosis and also exhibit other features in common with metastatic cancer cells [2]. Therefore, we first examined whether there was an imbalance between pro-apoptotic and anti-apoptotic genes in FLSs from RA patients compared with FLSs from OA patients. As shown in Figure 1A, the expression of anti-apoptotic Bcl-2 mRNA was higher in FLSs from RA patients while the expression of pro-apoptotic Bax mRNA was lower in FLSs from RA patients. Western blot analysis also showed similar results (Figure 1B). Using confocal microscopy, we identified the expression of Bcl-2 at the single FLS level. As shown in Figure 1C, Bcl-2 was highly expressed in RA FLSs. These findings verified that an imbalance between pro-apoptotic and anti-apoptotic genes exists in FLSs from RA patients.

We also evaluated whether the degree of apoptosis is different between FLSs in RA and OA. The results showed that in RA, FLSs were more resistant to SNP-induced apoptosis compared with FLSs in OA, which was determined by flow cytometry using PI and Annexin V (Figure 1D).

### IL-17 upregulates the expression of Bcl-2 in FLS from RA patients

**Figure 2:**
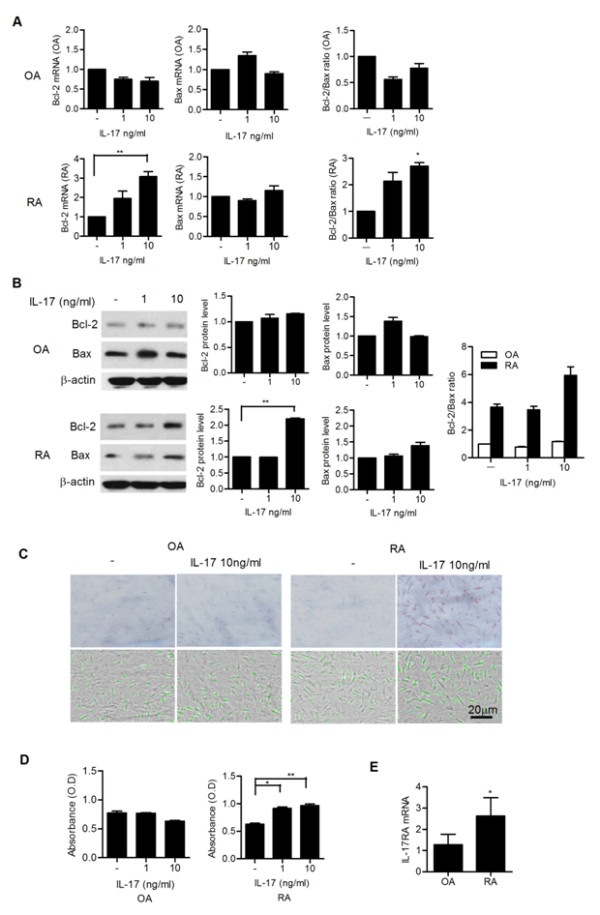
**Interleukin (IL)-17 upregulates the expression of Bcl-2 in synoviocytes in rheumatoid arthritis (RA)**. (**A**) Fibroblast-like synoviocytes from patients with RA (RA-FLSs) (*n *= 3) and fibroblast-like synoviocytes from patients with osteoarthritis (OA-FLSs) (*n *= 3) were cultured with IL-17 (0, 1 and 10 ng/ml) for 12 hours. The mRNA expression of Bcl-2 and Bax was evaluated by real-time PCR. The Bcl-2/Bax ratio is shown in the right panel. *P < 0.05, **P < 0.01 compared with the untreated group. (**B**) RA-FLSs (*n *= 3) and OA-FLSs (*n *= 3) were cultured with IL-17 (0, 1 and 10 ng/ml) for 12 hours. The expression of Bcl-2 and Bax was measured by western blot. The representative results are shown in the left panel. The Bcl-2/Bax ratio is shown in the right panel. **P < 0.01 compared with the untreated group. (**C**) RA-FLSs (*n *= 3) and OA-FLSs (*n *= 3) were cultured with IL-17 (0, 10 ng/ml) for 12 hours. FLSs were stained with anti-Bcl-2 antibody. Stained cells are shown in brown. (**D**) IL-17 promoted the proliferation of RA-FLSs, but not that of OA-FLSs. The proliferation of synoviocytes was determined using a CCK-8 kit as described in Materials and methods. *P < 0.05, **P < 0.01 compared with the untreated group. (**E**) The expression of IL-17RA mRNA in OA and RA FLSs was evaluated by real-time PCR. *P < 0.05 compared with OA.

We next investigated the effect of IL-17 on anti-apoptotic Bcl-2 expression in FLSs from RA patients. The results showed that treatment with IL-17 increased the expression of Bcl-2 in FLSs from RA patients both in mRNA and protein levels (Figure 2A and 2B). However, no significant effect of IL-17 on Bax expression was observed in FLSs from RA patients. Additionally, neither Bcl-2 nor Bax was changed by treatment with IL-17 in FLSs from OA patients.

We also investigated the expression of Bcl-2 in RA and OA FLSs by immunostaining. We found that intense Bcl-2 staining was observed in FLSs in RA compared with OA following IL-17 treatment (Figure 2C). We examined whether IL-17 affects the proliferation of FLSs from RA patients. The results showed that IL-17 promoted the proliferation of FLSs from RA patients by CCK-8 kit assay. However, IL-17 did not have a significant effect on the FLSs proliferation in OA patients (Figure 2D). Interestingly, we observed that IL-17RA was higher FLSs in RA than in OA (Figure 2E).

### STAT3 mediates IL-17-dependent Bcl-2 expression in FLSs from patients with RA

**Figure 3:**
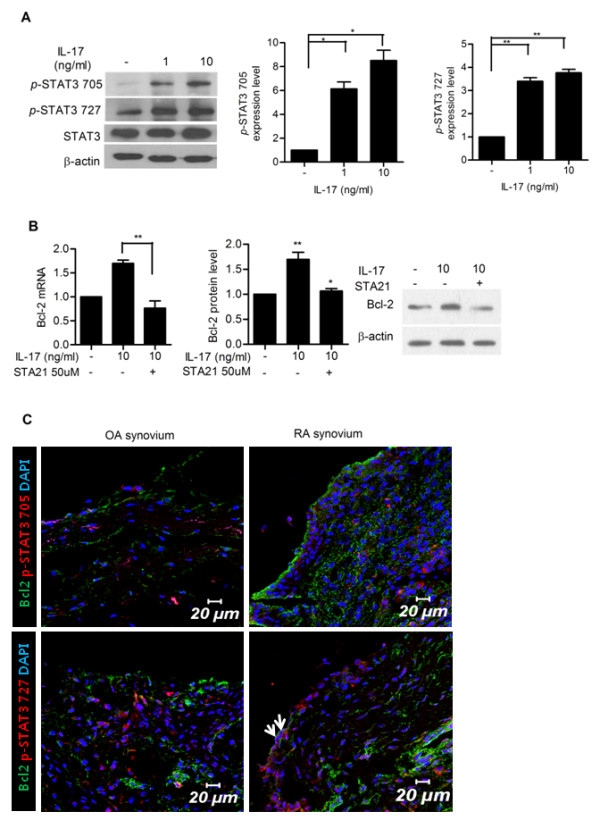
**Signal tranducer and activator of transcription 3 **(**STAT3) mediates IL-17-induced Bcl-2 upregulation in synoviocytes in rheumatoid arthritis (RA)**. (**A**) IL-17 upregulated the expression of STAT3 in RA-fibroblast-like synoviocytes (FLSs). RA-FLSs (*n *= 3) were cultured with IL-17 (0, 1 and 10 ng/ml) for 12 hours. The expressions of STAT3 and phosphorylated STAT3 (pSTAT3 705 and pSTAT3 727) were measured by western blot. The representative figure is shown in the left panel. Data are expressed as mean ± SD of three independent experiments. *P < 0.05, **P < 0.01 compared with the untreated group. (**B**) RA-FLSs (*n *= 3) were cultured with IL-17 (0 and 10 ng/ml) in the presence or absence of STAT3 inhibitor (STA21 50 μM) for 12 hours. The expression of Bcl-2 mRNA and protein was evaluated by real-time PCR and western blot. (**C**) Co-localization of STAT3 and Bcl-2 in rheumatoid synovium. Tissue sections from the synovium of patients with RA (*n *= 3) or osteoarthritis (OA) (*n *= 3) were stained with anti-Bcl-2, anti- pSTAT3 727 and anti-pSTAT3 705 antibodies. Co-localization of STAT3 and Bcl-2 was observed in synovial lining cells of RA patients. The representative figure is shown.

We examined whether IL-17 activates STAT3 in FLSs from RA patients. We observed that IL-17 treatment increased phosphorylated STAT3 (*p*-STAT3 705 and *p*-STAT3 727) in FLSs from RA patients, demonstrating that IL-17 activated STAT3 (Figure 3A). We also found that treatment with STA21, a STAT3 inhibitor, abrogated IL-17-induced Bcl-2 expression in FLSs from RA patients (Figure 3B), demonstrating that STAT3 is involved in IL-17-induced Bcl-2 expression. When we cultured FLSs from RA patients with STA21 in the absence of IL-17, STA21 did not cause significant impact on the expression of Bcl-2 (data not shown).

To verify this observation *ex vivo*, immunohistochemical staining for phosphorylated STAT3 (*p*-STAT3 705 and *p*-STAT3 727) and Bcl-2 was conducted on the synovia of RA and OA patients. As a result, Bcl-2 and phosphorylated STAT3 (*p*-STAT3 727) colocalize in arthritic joint tissues of RA patients, particularly in the lining layer of the synovium (Figure 3C).

### IL-17 promotes the proliferation of synoviocytes and rescues the synoviocytes from apoptotic death induced by SNP via STAT3 pathway

**Figure 4:**
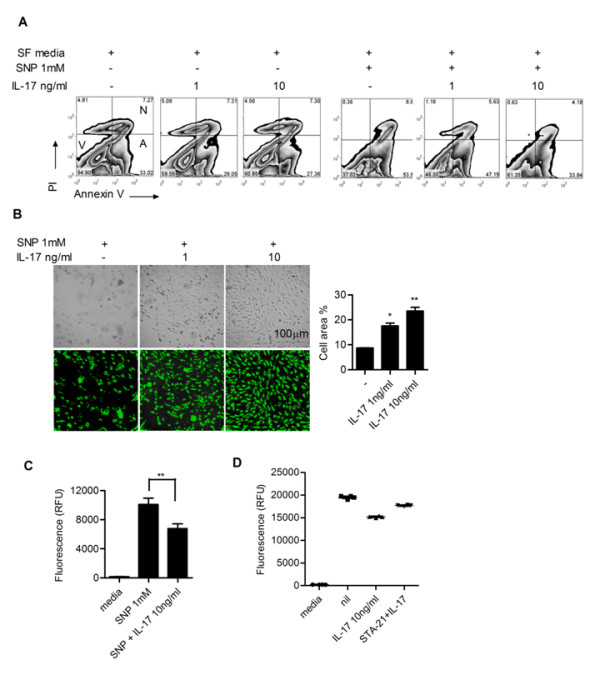
**IL-17 promotes the proliferation of synoviocytes and rescues the synoviocytes from apoptotic death induced by sodium nitroprusside (SNP) via the STAT3 pathway**. (**A**) IL-17 protects synoviocytes from apoptotic death. RA- fibroblast-like synoviocytes (FLSs) (*n *= 3) were cultured with IL-17 (0, 1 and 10 ng/ml) in the presence or absence of 1 mM SNP in serum-free DMEM for 24 hours. The degree of apoptosis was assessed by flow cytometry using propidium iodide (PI) and Annexin V. Representative results are shown. (**B**) Phase contrast microscopy showed that treatment with IL-17 dose-dependently rescued synoviocytes from apoptotic death induced by SNP. The percentage of cell area was determined using the TOMORO analySIS TS Lite Image program (Olympus, Münster, Germany). Data are expressed as mean ± SD. *P < 0.05, **P < 0.01 compared with untreated group. (**C**) Apoptosis activity was assayed using a caspase 3/7 assay kit and a fluorescence luminometer. Data are expressed as mean ± SD. **P < 0.01 compared with the SNP-treated group. (**D**) Apoptosis activity was indicated by fluorescence using a caspase3/7 assay kit. The results of three individual experiments are shown.

We finally investigated the impact of IL-17 on the survival and proliferation of FLSs from RA patients. FLSs from RA patients demonstrated low rate spontaneous apoptosis (data not shown). SNP (1 mM) induced significant apoptosis of FLS, which was determined by calculating PI-negative Annexin V-positive cells by flow cytometry. As shown in Figure 4A, IL-17 protects the synoviocytes from apoptotic death induced by SNP as in a previous report [18]. Figure 4B and Figure 4C show that treatment with IL-17 dose-dependently rescues the synoviocytes from SNP-induced apoptotic death. Interestingly, treatment with a STAT3 inhibitor (STA21) reversed the anti-apoptotic effect of IL-17 on FLSs, thus verifying that STAT3 mediated IL-17-mediated survival of FLSs from RA patients (Figure 4D).

## Discussion

Human IL-17 was first cloned from a CD4+ T cell library in 1995 [19] and it is an important pro-inflammatory cytokine. Both Th17 cells, the major producer of IL-17, and IL-17 play an important role in the pathogenesis of a diverse group of immune-mediated diseases, including psoriasis [20,21], multiple sclerosis [22], inflammatory bowel disease [23] and asthma [24]. Data obtained from humans and mice demonstrated that IL-17 is critically implicated in the pathogenesis of RA. A single injection of IL-17 into a normal mouse knee joint induces inflammatory arthritis [25]. IL-17-knockout mice develop significantly less severe arthritis than do wild-type mice, and treatment with neutralizing IL-17 antibodies or soluble IL-17 receptor attenuates joint inflammation [26-28]. In humans, IL-17 increases in the sera and synovial fluid of RA patients and is highly expressed in the rheumatoid synovium [29,30].

Synoviocytes are the major cell population of inflamed synovial tissue of RA patients and are found to be a critical component for the development of RA. With regard to the effects of IL-17 on synoviocytes, much effort has been made to identify the impact of IL-17 on synoviocytes. IL-17 alone induces the production of IL-6 and IL-8 in FLSs from RA patients [31]. IL-17 can synergize with other pro-inflammatory cytokines such as TNF-α and IL-1β and thereby promotes cytokine production in FLSs from RA patients [32,33]. In addition, IL-17 contributes to inflammatory cell infiltration into inflamed joint tissues by inducing the production of chemokines, such as IL-8 and stromal cell-derived factor-1 (SDF-1) in FLSs from RA patients [12,34]. It also stimulates the release of degradative enzymes by synoviocytes, promoting synovium, ligament and cartilage matrix destruction. Moreover, IL-17 is able to promote the expression of receptor activator of nuclear factor-κB (NF-Kb) ligand (RANKL), the leading player in osteoclastogenesis, in synoviocytes and osteoblasts, which contribute to the bone destruction that is closed related to functional disability in RA patients [35]. Based on evidence suggesting that IL-17 is critically involved in the pathogenesis of RA, clinical trials of IL-17-targeted therapy are underway in patients with RA [36,37].

Until now, only a few reports have investigated the effect of IL-17 on the survival or proliferation of rheumatoid synoviocytes [18,38]. Zhang *et al*. demonstrated that IL-17 promotes the proliferation of FLSs from RA patients and that Cyr61 is deeply implicated in this event [38]. Toh *et al*. reported that IL-17 prolongs the survival of FLSs by regulating synoviolin expression [18].

STAT3 is an essential transcription factor in cellular physiology. It is activated through phosphorylation of tyrosine 705 and serine 727 in response to various cytokines and growth factors including interferons, epidermal growth factor, IL-5, IL-6, IL-21, hepatic growth factor, leukemia inhibitor factor, and bone morphogenic protein 2, and also the hormone leptin. STAT3 mediates the expression of a variety of genes in response to cell stimuli, thus, playing a key role in many cellular processes such as cell growth and apoptosis. One report has shown that survival of rheumatoid synoviocytes is dependent on STAT3 [39]. It is well-known that STAT3 is critical in the differentiation of Th17 cells, the major IL-17 producing cells [14]. In contrast, only one report has indicated that STAT3 is downstream of IL-17 signaling in human airway smooth muscle cells [40]. Our results demonstrated for the first time that STAT3 is critical in IL-17 signaling in patients with RA. Thus, a strategy targeting STAT3, which is critical both in the differentiation of IL-17-producing Th17 cells and IL-17 signaling, might be a good therapeutic tool to treat patients with RA.

In the current study, we used STA21 as a STAT3 inhibitor. STA-21 is a small molecule with potent STAT3-inhibiting activity. It impedes STAT3 DNA binding activity, STAT3 dimerization and STAT3-dependent luciferase activity. It also inhibits breast cancer cells that express constitutive active STAT3 [41].

In this study, IL-17 increased Bcl-2 expression and promoted the proliferation FLSs in RA patients, but not in OA patients (Figure 2A, B and 2D). Therefore, it is conceivable that FLSs from RA patients are more responsive to IL-17 stimulation than those from OA patients. These findings might be partially explained by the differential expression of the IL-17 receptor between FLSs from RA patients and FLSs from OA patients [42]. Our results also showed that the expression of IL-17 receptor (IL-17RA) was higher in FLSs from RA patients than in those from OA patients (Figure 2E).

In the present study, we found that pro-apoptotic Bax gene is decreased and anti-apoptotic Bcl-2 gene increased in FLSs from RA patients compared with those from OA patients. IL-17 upregulated the expression of Bcl-2 in FLSs from RA patients, but not in FLSs from OA patients. Additionally, STAT3 mediated IL-17-induced Bcl-2 upregulation in FLSs from RA patients. Moreover, we observed that IL-17 promoted the proliferation of synoviocytes and rescued the synoviocytes from apoptotic death via the STAT3 pathway. Taken together, these findings suggest that the IL-17/STAT3 pathway is essential for the survival and proliferation of synoviocytes.

## Conclusions

In conclusion, the present study demonstrated that STAT3 is critical in IL-17-mediated survival of FLSs in patients with RA. Our data underline the importance of the IL-17/STAT3 pathway as a strong potential candidate for therapeutic modulation of RA.

## Abbreviations

DAPI: 4',6-diamidino-2-phenylindole; DMEM: Dulbecco's modified Eagle's medium; ECL: enhanced chemiluminescence; ELISA: enzyme-linked immunosorbent assay; FCS: fetal calf serum; FLS: fibroblast-like synoviocytes; HRP: horseradish peroxidase; IL: interleukin; IL-17RA: interleukin-17 receptor A; OA: osteoarthritis; PCR: polymerase chain reaction; PI: propidium iodide; RA: rheumatoid arthritis; SDF-1: stromal cell-derived factor-1; SNP: sodium nitroprusside; STAT3: signal tranducer and activator of transcription 3; TBS: Tris-buffered saline; Th17: T-helper 17; TNF-α: tumor necrosis factor- α; TTBS: Tris-buffered saline with 0.1% Tween 20; VEGF: vascular endothelial growth factor.

## Competing interests

The authors declare that they have no competing interests.

## Authors' contributions

SYL, SKK, MLC and HYK contributed to the conception and design, acquisition of data, analysis and interpretation of data, and drafting of the article. HJS and JGR performed all the experiments and participated in drafting the manuscript. EKK and HJO contributed to acquisition of data and immunohistochemical staining. JHJ and SHP contributed to conception and design, and drafted the manuscript. All authors read and approved the final manuscript.
